# GPFrontend and GPGraphics: graphical analysis tools for genetic association studies

**DOI:** 10.1186/1471-2105-11-472

**Published:** 2010-09-21

**Authors:** Steffen Uebe, Francesca Pasutto, Mandy Krumbiegel, Denny Schanze, Arif B Ekici, André Reis

**Affiliations:** 1 Institute of Human Genetics, University of Erlangen-Nuremberg, Schwabachanlage 10, 91054 Erlangen, Germany; 2 Institute of Human Genetics, University of Magdeburg, Leipziger Straße 44, 39120 Magdeburg, Germany

## Abstract

**Background:**

Most software packages for whole genome association studies are non-graphical, purely text based programs originally designed to run with UNIX-like operating systems. Graphical output is often not intended or supposed to be performed with other command line tools, e.g. gnuplot.

**Results:**

Using the Microsoft .NET 2.0 platform and Visual Studio 2005, we have created a graphical software package to analyze data from microarray whole genome association studies, both for a DNA-pooling based approach as well as regular single sample data. Part of this package was made to integrate with GenePool 0.8.2, a previously existing software suite for GNU/Linux systems, which we have modified to run in a Microsoft Windows environment. Further modifications cause it to generate some additional data. This enables GenePool to interact with the .NET parts created by us. The programs we developed are GPFrontend, a graphical user interface and frontend to use GenePool and create metadata files for it, and GPGraphics, a program to further analyze and graphically evaluate output of different WGA analysis programs, among them also GenePool.

**Conclusions:**

Our programs enable regular MS Windows users without much experience in bioinformatics to easily visualize whole genome data from a variety of sources.

## Background

Besides genotyping individual DNAs in whole genome association studies, the use of pooled DNA has recently become a feasible alternative. This is based on the assupmtion that hybridization intensities at single array oligonucleotides of the respective alleles should reflect allelic frequencies in the DNA pool [1]. Pooling-based SNP array approaches have successfully been used in a wide range of studies, such as the identification of potential risk factors for alcohol addiction [2], mental impairment [3] or abnormal cholesterol levels [4]. Despite recent developments in array technology, the use of pooled DNA still outperforms single DNA genotyping in terms of cost efficiency [5]. As pooling-based studies are a relatively new method, a broad spectrum of analysis software, as it is available for many other applications, is still missing. The primary software project previously dedicated to this purpose [6] was originally intended for GNU/Linux systems and therefore not usable on Microsoft Windows, which is, however, ubiquitous even in the laboratory environment. Additionally, as with many Linux centered software products, it relied entirely on text configuration files, command line options and text output; graphical evaluation of the data is still not intended. There is another software package, MPDA [7], which was recently developed using MATLAB; it is, however, dependent on a complete MATLAB installation for the GUI version, or, for the command line version, on a MATLAB runtime environment.

## Implementation

### GenePool

Version 0.8.2 of the GenePool software [6] has been used to study complex traits, such as Schizophrenia [8] or progressive supranuclear palsy [9]. The software most prominently contains two modules: *gpextract *extracts intensity values from the data files of the chip system (Affymetrix CEL files or Illumina text files) and stores them in a smaller binary format. Furthermore, it creates text metafiles pointing to those binary files. Downstream analysis is done using *gpanalyze*, which calculates relative allele signal (RAS) values from the intensities and performs various calculations with those. The output of *gpanalyze *consists of a number of text files. The GPFrontend and GPGraphics user manual, which is included with the program files (Additional file 1), contains a graphical representation of the GenePool workflow.

After adjusting some code of both *gpextract *and *gpanalyze *to enable them to run in a Microsoft Windows environment, we further modified *gpanalyze *in a way that one of its output files can be more productively used for analysis in GPGraphics. The first modification was the adding of some code to calculate the overall difference in mean RAS values, preserving the sign in the process, and write the results to a text file. The second modification causes gpanalyze to additionally include both this calculated mean RAS difference and the rank value for each SNP in its output. The output file in question has the SNPs sorted by chromosome and position, so it can immediately be used as input for GPGraphics.

### GPFrontend

In order to facilitate the use of *gpextract *and *gpanalyze*, a graphical user interface was created. Here, the user may select the input files and specify some additional options. Upon confirmation by the user, *gpextract *is called for every single input data file. At the same time, a text file is created by GPFrontend, which references the text files produced by *gpextract*. This file is necessary for *gpanalyze *to process the data further.

GPFrontend can also be used to merely generate the metadata files for already extracted SNP data, e.g., when comparing several different combinations of the same data set. Thus, the extraction process only has to be done once for every SNP data file, and many of the text files normally generated by *gpextract *can instead be quickly produced by GPFrontend. A step-by-step tutorial showing how to use GPFrontend and GPGraphics to analyze pooling data is provided with this article (Additional file 2, Tutorial 1).

Both *gpextract *and *gpanalyze *have a large number of command line options, the latter with its various and flexible algorithms even more than the former. The motivation to use a graphical interface especially with gpanalyze is therefore high. The frontend for *gpanalyze *enables the user to see and manipulate all possible options at once. Upon confirmation by the user, *gpanalyze *is called with the selected options.

### GPGraphics

**Figure 1:**
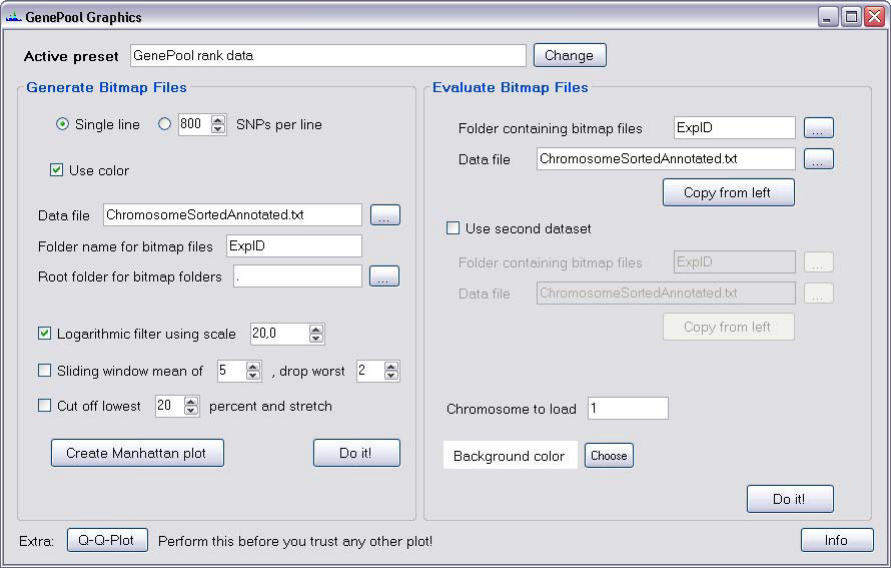
**The main window of GPGraphics**. Here, several options regarding the generation of bitmap files, mathematical filters and the input files are selected. Data from the left side can simply be copied to the right, so that images can be viewed right away.

One of the major disadvantages of GenePool is its complete lack of any graphical output. To overcome this, GPGraphics (Figure 1) was originally developed to read one of the output files of *gpanalyze *and create a PNG compressed bitmap file for every chromosome. In those files, the scores or ranks of all analyzed SNPs are shown as lines of corresponding height.

Additionally, GPGraphics writes a text data file for every Chromosome, containing the numerical heights of the lines in the bitmap file. The researcher is thus not limited to visual analysis of the data, but can also find loci by defining strict criteria and applying these to the data files using third party software, such as a spreadsheet program.

**Figure 2:**
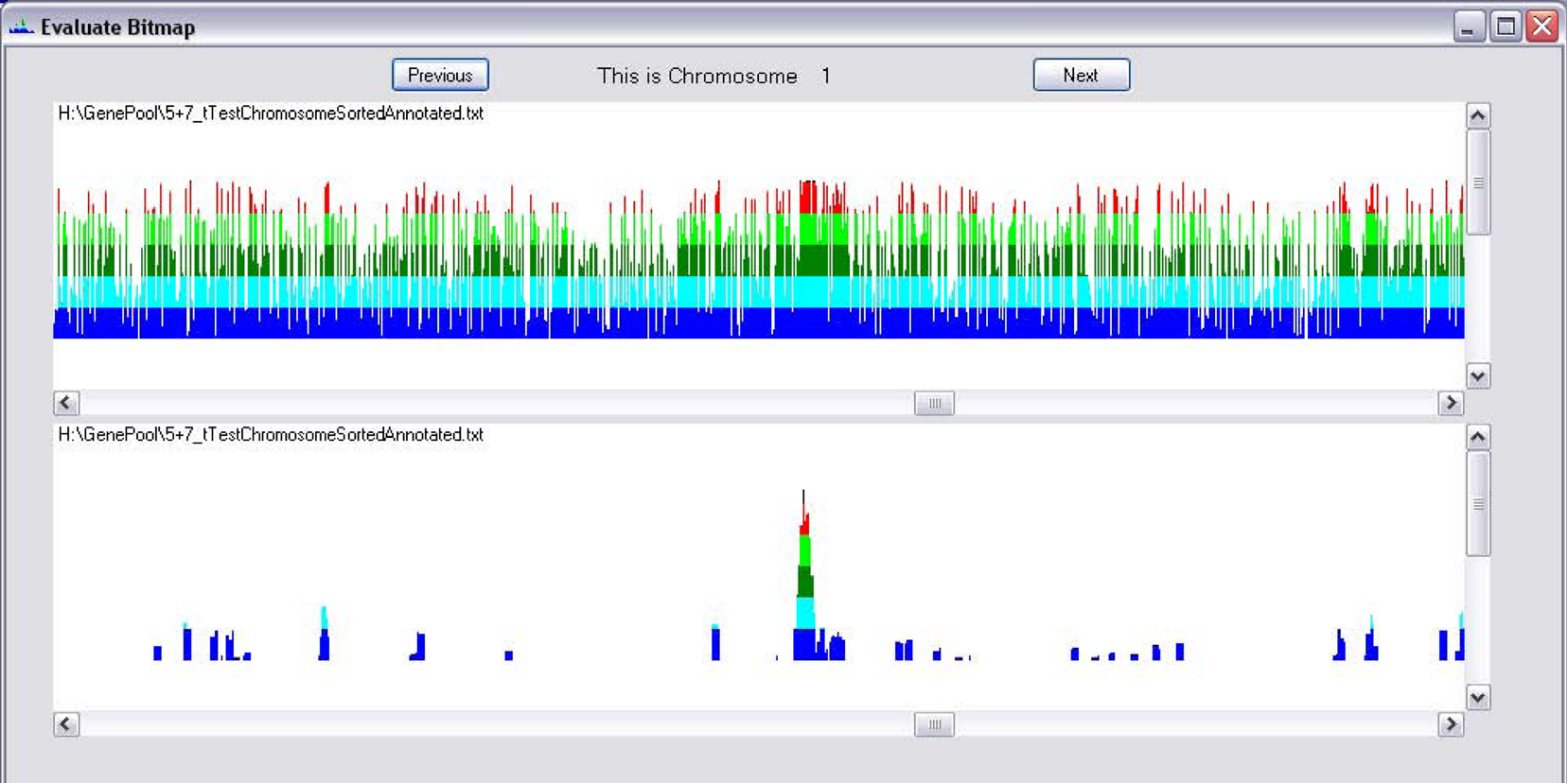
**Effect of Filters on Visibility of Peaks**. The upper pane shows an unfiltered rank plot of T-test values from a pooling experiment. In the lower pane, a logarithmic transformation with *f *= 50, a sliding window mean with *m *= 5 and *n *= 0 and a cutoff of the lowest 20% have been performed in that order; the peak comes out quite clearly after this.

As there is usually a lot of background noise in the data (Figure 2), it may sometimes be quite difficult to discern regions with promising SNPs from others. Therefore, some filter functions are incorporated into GPGraphics: There is a logarithmic filter, which assigns new values based on the original ones and an exponential curve by performing the following transformation:

$$r_{new}=\frac{2^{\frac{rf}{n}}n}{2^{f}}$$

where *n *is a scaling factor depending on what values the graphical output expects. In GPGraphics, the graphical output function takes input values from 0 to 100, so 100 represents a fully saturated signal. Therefore, *n *is set to 100 in GPGraphics. *f *is a user-selectable factor determining the stringency of the filter; *r *and *r*_*new *_are the old and the new score, rank or log_10_(p-value) for that SNP, respectively. The highest values will appear enhanced, while the background will be significantly reduced without being completely eliminated. Furthermore, a variable sliding window mean calculation can be performed, assigning to every SNP the average of the scores of the best *m *- *n *out of itself and the following *m *- 1 SNPs (*m *and *n *are user-selectable). Finally, there is also a simple cutoff function, which will crop the lowest *p *percent of the maximum value and "stretch" the remainder to normalize to 100%.

For a quick overview of the entire data set, an unfiltered "Manhattan" plot can also be generated either from the input file as selected in the main window or from a separately specified file. GPGraphics can handle any type of tab or space delimited text file, with or without a header line. Number and content of the data columns and the maximum expected value are specified by the user, with presets for GenePool and several other analysis programs (e.g. PLINK, EIGENSTRAT) already implemented.

The generated bitmap files (not the Manhattan plot) may also be viewed and evaluated in GPGraphics, in which case the user can choose between looking at a single dataset or two datasets (necessarily from the same chip type) displayed simultaneously. The second option is useful when comparing different experiments or filter methods. By clicking on any of the SNP lines, the user gets a set of information about the selected SNP. Due to our modifications to gpanalyze, this information also includes the difference of the mean RAS values of cases and controls, preserving the sign. Thus, it is evident which allele is predominant in cases or controls. The SNP information can then be copied to the clipboard or submitted to the UCSC Genome Browser (http://genome.ucsc.edu/cgi-bin/hgGateway as of 2010-09-20) to view the genomic region of the selected SNP.

**Figure 3:**
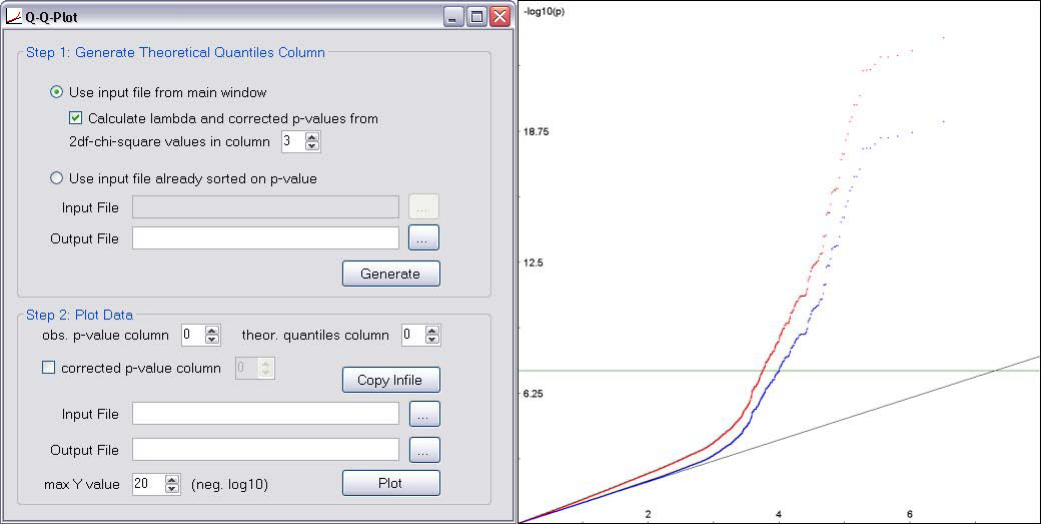
**Q-Q plot module of GPGraphics**. On the left, the user interface of the module. In the top part, a file for plotting can be generated from sorted or unsorted output. In the bottom part, columns to plot can be selected from a file. On the right, an example Q-Q plot.

GPGraphics also includes a module to generate a quantile-quantile plot of expected versus observed p-values (Figure 3). The p-values of the input file are sorted and an expected quantiles column is added. If the values are from a χ²-test with 2 degrees of freedom, an additional column with a GC-corrected p-value [10] based on the median χ² score may be created and included in the Q-Q-plot.

## Results and Discussion

Originally intended as a graphical extension of an analysis software for pooling based studies, especially GPGraphics has developed into a universal visualization tool for the output of many different analysis programs, such as PLINK or EIGENSTRAT. Since it can be easily customized to accommodate almost any text output sorted in map order, it enables even inexperienced users to get an overview of whole genome association data and to visually search for loci warranting further study. When analyzing data from a single sample GWAS, such as PLINK output, the logarithmic and sliding window filters will not normally be used. Nonetheless, the ability to visually browse through the entire genome data set while seeing every single SNP raises the chances of spotting interesting loci, which might have been lost in a purely algorithmic, non-visual analysis. In a tutorial document provided with this article (Additional file 2), we show how this has been done in an actual GWAS (Tutorial 2). Especially smaller research institutions, who may not have the funds nor the bioinformatics personnel required for a large scale association study are now able to profit from the benefits of whole genome microarrays. A pooling based study with a small number of microarrays used can be easily evaluated with GenePool, GPFrontend and GPGraphics on a Windows PC, thus providing valuable hints to, e.g., new candidate regions for functional studies.

Even if the data are very noisy, as is often the case in pooling based studies, the implemented filters enable researchers to pick up signals that would possibly be overlooked when analyzing the raw data. In the tutorials provided in Additional file 2, this is shown using actual data from a pooling study (Tutorial 1). Relative freedom in trying out different filters and their combinations is justified, as the main purpose of the graphical evaluation is the search for sites to be investigated further.

Finally, the Q-Q-plot module enables even researchers with little background in bioinformatics or biostatistics to generate a plot of their data from which the reliability of the results can easily be judged. For this, we also provide a tutorial (2a) in Additional file 2. Currently, q-q plots only work with p value data, but an extension to score data is planned for a future release.

## Conclusions

We have created an easy-to-use program for visually analyzing whole genome association data, especially from pooling based approaches. With this software, many researchers with regular desktop PCs and no bioinformatics personnel will now be able to use and profit from whole genome association data nonetheless. With its small size, our software is easy to obtain and install.

## Availability and requirements

The most recent versions of GPFrontend and GPGraphics are contained within this article (Additional file 1 - supp1.zip).

Operating systems: Platform independent; tested on Windows (XP, Vista and 7) and GNU/Linux (Open SuSE 11.1, 11.2 and 11.3)

Programming language: Visual Basic .NET

Hardware requirements: RAM usage depends on the size of the input files; for whole genome association data, at least 1 GiB is recommended

Other requirements: An implementation of the .NET 2.0 runtime (integral part of Windows Vista and Windows 7; must be installed on Windows XP and 2000) or the mono runtime (with package mono-basic)

License: Closed source, free for non-commercial use.

## Authors' contributions

SU designed and programmed the software and wrote the manuscript. FP provided the original idea and specifications and revised the manuscript. MK, DS and ABE shaped the development of the software by using it and requesting new features as needed. AR revised the manuscript and had the overall responsibility of the projects from which the development of the software sprang. All authors read and approved the final manuscript.

## Supplementary Material

Additional file 1 **GPFrontend and GPGraphics**. GPFrontend.exe, GPGraphics.exe with GPGPresets.ini (presets for different input file formats), GPManual.pdf (a user manual for GPFrontend and GPGraphics) and a README file. Click here for file

Additional file 2 **Tutorials for GPFrontend and GPGraphics**. Three step-by-step tutorials with actual data for both pooling based studies and single sample GWAS Click here for file
