# Supplementary material for: GPFrontend and GPGraphics: graphical analysis tools for genetic association studies
Source: BMC Bioinformatics. 2010 Sep 21;11:472. doi: 10.1186/1471-2105-11-472 (PMC2949897; doi:10.1186/1471-2105-11-472)
Supplement: Additional file 1 — GPFrontend and GPGraphics. GPFrontend.exe, GPGraphics.exe with GPGPresets.ini (presets for different input file formats), GPManual.pdf (a user manual for GPFrontend and GPGraphics) and a README file. [file 1471-2105-11-472-S1.ZIP › GPManual.pdf]

GPFrontend and GPGraphics

User Manual

Introduction

GPFrontend and GPGraphics were originally developed to provide a user-friendly front- and backend to the GenePool software by the Translational Genomics Research Institute in Phoenix, Arizona.

GPFrontend is mainly a graphical user interface to the two parts of GenePool: *gpextract* and *gpanalyze*. Additionally, it creates metafiles required by *gpanalyze*, enabling analyses using different parameters without the need of new runs of *gpextract*.

GPGraphics is a universal graphical backend originally developed for the analysis of GenePool output. It can, however, be used to graphically evaluate whitespace-delimited text output files of any platform.

GenePool

GenePool 0.8.2 (the current stable version) consists of two modules: *gpextract* extracts the signal intensities from Affymetrix or Illumina intenstiy files into smaller and more manageable binary files. Case or control status of a sample is determined by user input at this stage. Metafiles are created, which reference the binary files as cases or controls. The binary files (.gpb) can then be used by *gpanalyze* to perform a variety of statistical tests.

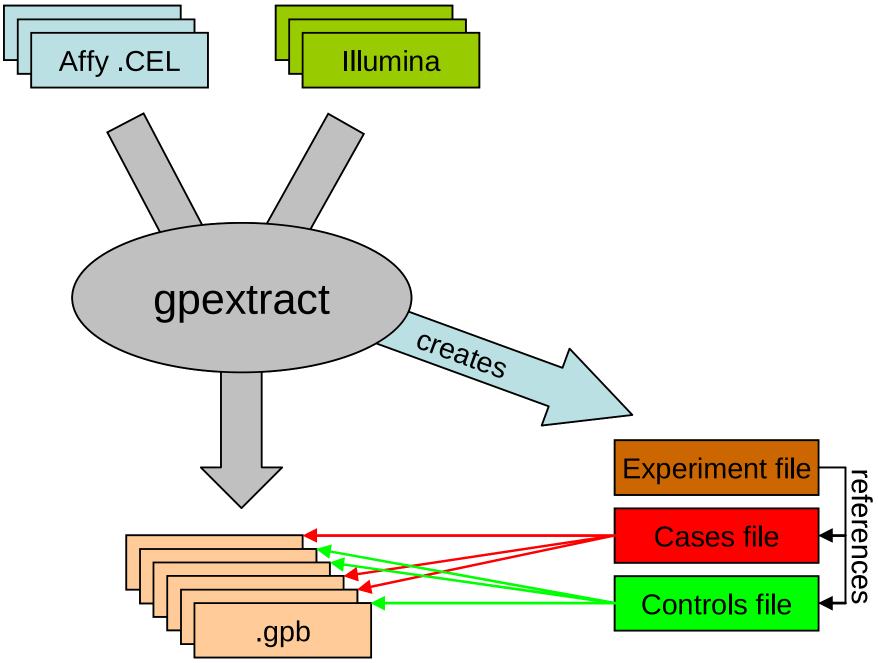

Step 1: *gpextract* extracts intensity values from Affymetrix or Illumina data and creates metafiles referencing the extracted files as either cases or controls.

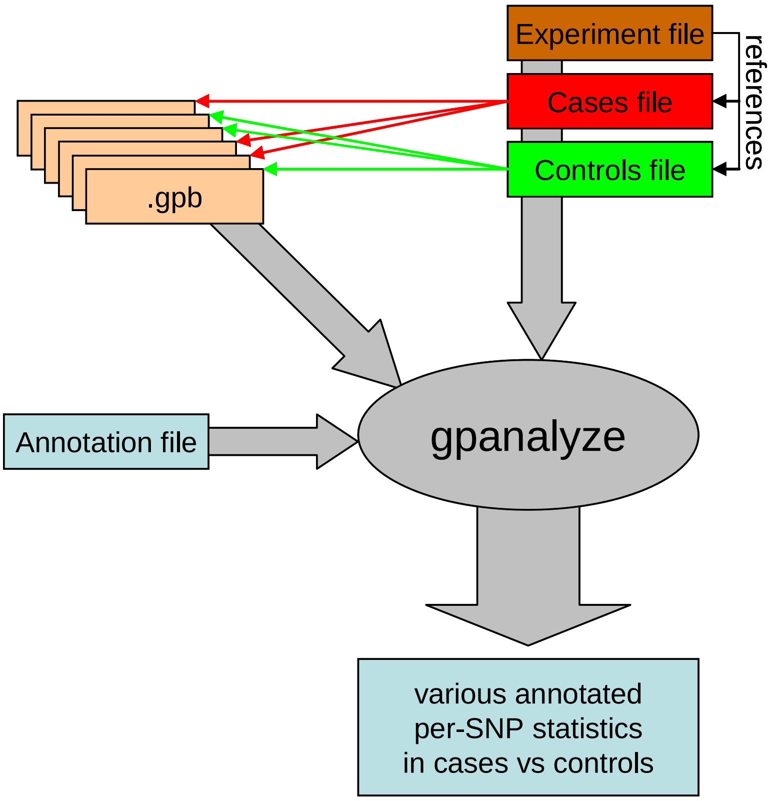

Step 2: *gpanalyze* reads the metafiles and the case and control intensity files referenced by them. Statistical tests (silhouette score, t-test and others) can then be computed for each SNP. Annotation data (Chromosome and Position) are added.

Both programs are called via command line. As gpextract will only process one intensity file at a time, it must be called sepearately for every such file. Final results are stored in several text files, containing data for one SNP per line.

GPFrontend

From the main window of GPFrontend, the user can choose the module to use. The first step will usually be “Use gpextract”.

The gpextract frontend window has two list boxes, to which intensity files can be added with the “Add” button (multiple files at once may be selected). The Enzyme used in the chip chemistry should also be selected. The chip layout file (.CDF file) must be provided; those files can be downloaded from Affymetrix’ web site. The default name for the master metafile to be used in gpanalyze later on is “Experiment.txt” , a different name can, however, be selected here.
On clicking “Do it!”, GPFrontend will call gpextract with the provided options for every intensity file. At this point, if all .CEL files have been processed, the gpanalyze frontend can be called to finalize the analysis.

Support for Illumina files is implemented in GPFrontend, but has yet to be tested and is therefore temporarily disabled.

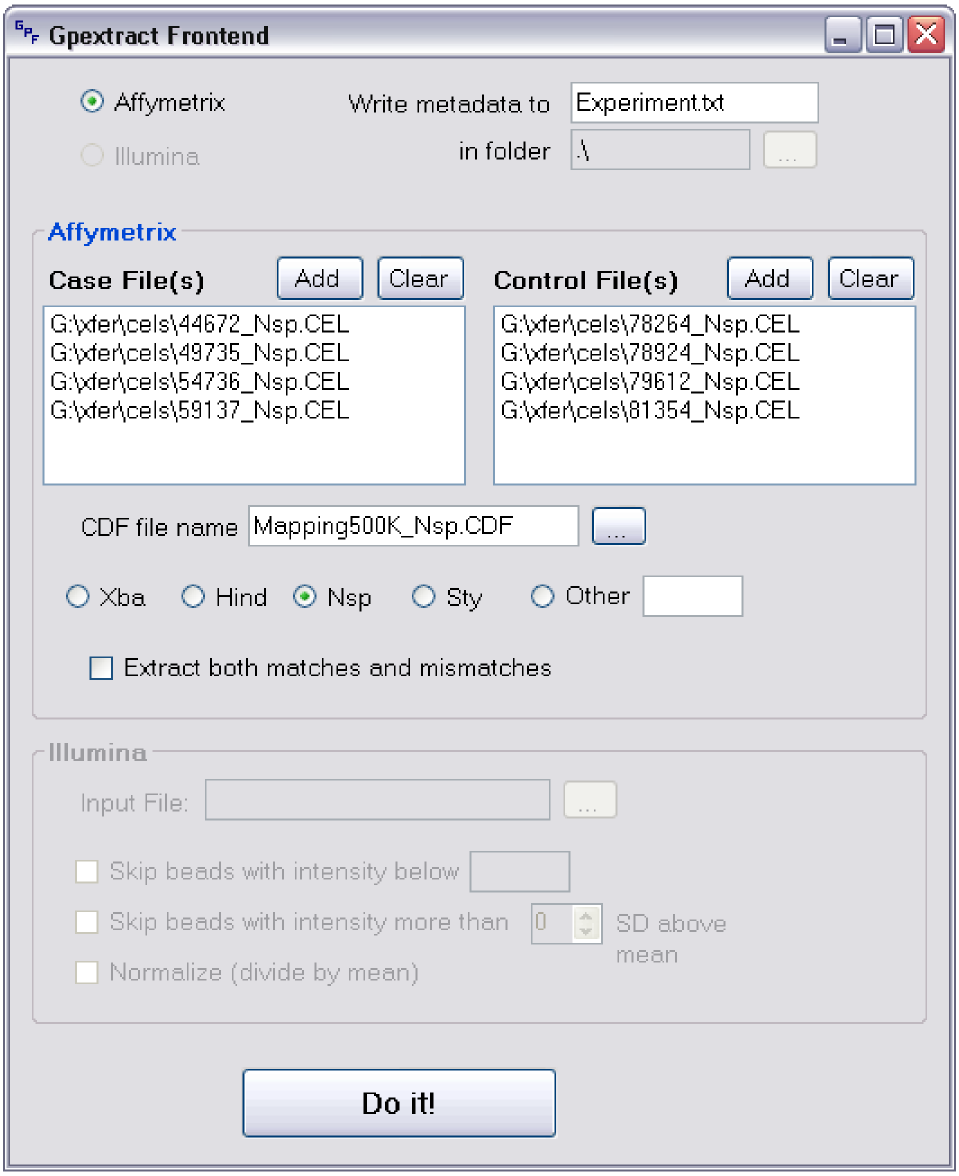

The interface to call gpextract. On “Do it!”, GPFrontend will call gpextract once for every .CEL file in the lists and then generate the metafiles required by gpanalyze.

The next logical step would now be to call gpanalyze, using the frontend provided for that purpose. The many available options of gpanalyze are not covered in any detail here, as they are described in the documentation for GenePool.

All that gpanalyze needs to be told about the files extracted in the previous step is the name and location of the master metadata file (usually “Experiment.txt”).

An annotation file containing probe ID, dbSNP rsID, chromosome and physical position of every SNP on the array should be provided here. Annotation data is also available for download from Affymetrix.

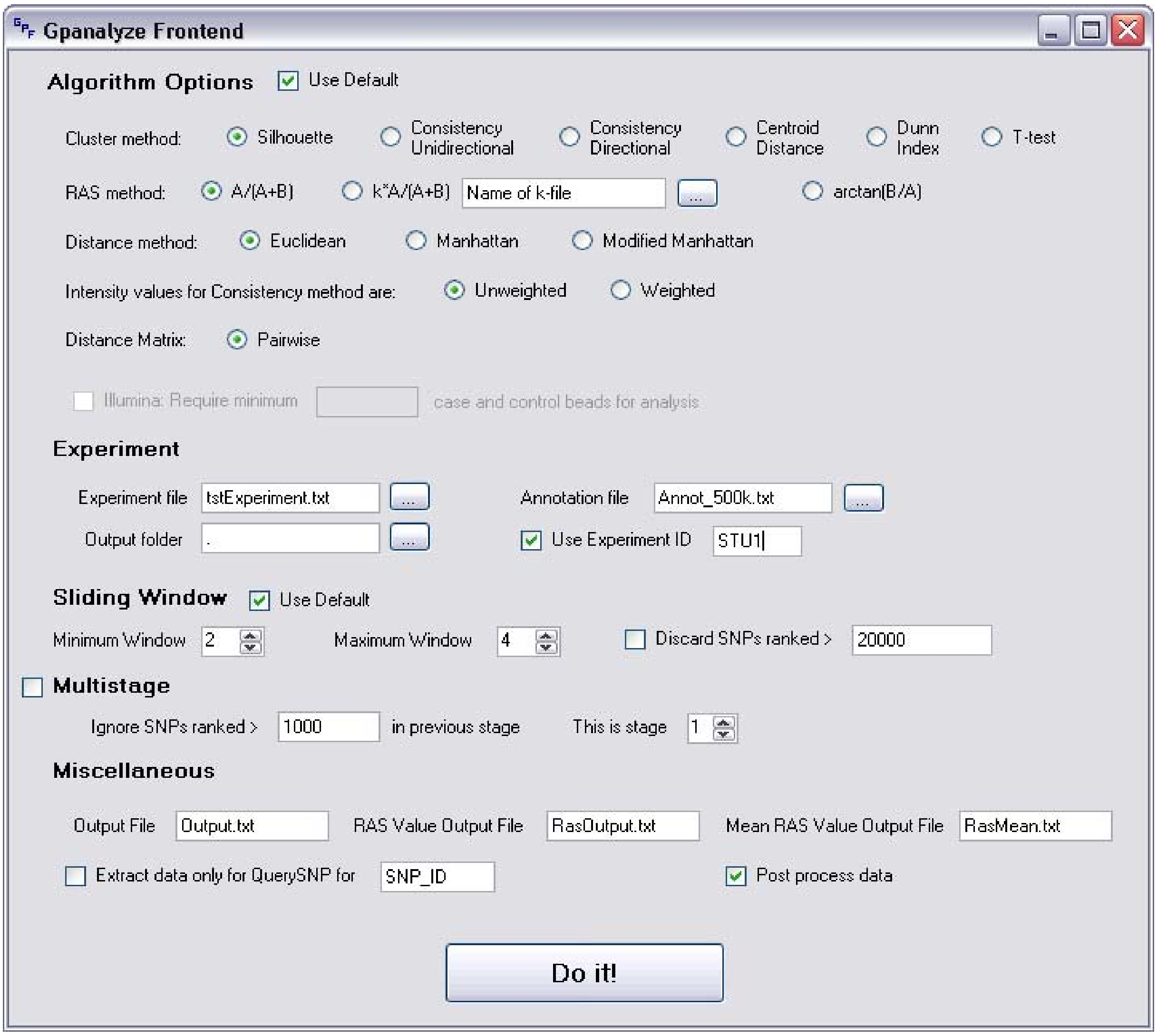

The frontend for gpanalyze, showing all available options. On “Do it!”, gpanalyze is called, taking input as specified in the “experiment file”, i.e. the master metadata file.

Sometimes it is desirable to re-analyze already extracted data (.gpb files) in a different combination than the one specified at the time of extraction.

To facilitate this, GPFrontend has a module to generate metadata for already extracted intensity files. The interface is essentially the same as in the gpexract frontend. The main differences are:

- extracted files (.gpb) are selected instead of raw intensity files
- for a two-enzyme array (such as the Affy 500k), files for both enzymes can be entered at the same time
- a .CDF file is not needeed.

So once the intensities are extracted into the .gpb files, these can now be added to the case or control lists as the experiment requires; in the above example, one could now test the four case files (44672 to 59137) versus only three of the controls, simply by adding only those 3 to the control lists and then generating new meta data.

GPGraphics

GPGraphics provides four main functions:

- generating images vor interactive viewing
- viewing such images interactively
- generating genome wide “Manhattan” plots
- as an extra: calculate expected quantiles and create a quantile-quantile (QQ) plot

GPGraphics accepts as input almost any whitespace (i.e. tab or space) delimited text file, with or without a header line. Multiple adjacent separators, such as the spaces in fixed-width files, will be treated as one.

The following information about the input file must be provided:

- presence or absence of a header line
- if no header, names of the columns
- number of columns
- which columns contain chromome, physical position and data
- type of data: p-value, odds ratio or score
- expected maximum value for data, i.e. upper threshold for graphics

The first four are rather self-explanatory. The type of data determines the way the plots are generated: for “score” type data, from the value itself, for “p-value” type data from the negative decadic logarithm of the value; and for “odds ratio” type data, from the absolute value of the dyadic (i.e., base two) logarithm. Rank data will be plotted with rank 1 as the highest peak.

In order to scale the plots properly, the expected maximum value must be given. The value entered here will correspond to a peak height of 100 pixels, or five color bands, in the plot. For p-values and odds ratios, the appropriate transformations are made. So, setting the expected maximum value to 10 and the data type to “p-value” would mean that a peak of 100 pixels in the plot corresponds to a p-value of 10E-10. Rank data, of course, needs no such preset maximum.

For convenience, several presets for output files of common programs, including GenePool, are provided. They are stored in GPGpresets.ini, along with instructions how to include new presets. The currently active preset is shown in the topmost text box of the main window. It can be changed to another preset or to custom settings.

Generating images for interactive viewing

The left part of the main window (“Generate Bitmap Files”) is dedicated to this task. The format of the plot must be chosen; either a fixed number of SNPs per row or all SNPs in one row (which effectively means up to 30,000 in a row due to operating system constraints). If a color plot is desired, which will usually be the case, every peak of the plot will be divided into evenly spaced color bands, a change in color occurring every fifth of the expected maximum value.

GPGraphics contains various mathematical filters to better visualize signals in noisy data, e.g. from DNA pooling based experiments. These filters are described in detail in the publication of which this software is part.

There is a logarithmic filter, which is especially useful for rank data, as it will enhance the contrast between higher and lower values. The sliding window mean filter will do just that – calculating the mean value in a sliding window of n SNPs, dropping the lowest m values altogether. Finally, a variable cutoff can be applied, after which the remainder (above the threshold) will be stretched accordingly. So if, for example, the lowest 20% of a plot are cut off, the remaining 80% are stretched to fill the space of the original 100%.

After file names and locations have been entered, the images and accompanying data files (“.dat”) for each chromosome can be generated by clicking “Do it!”. The data files contain the actual calculated height of each data peak as a non-integer value, for possible later evaluation, such as in a spreadsheet program.

There is also a Button to create a Manhattan plot, so as to get a quick genome wide overview of the top hits .

Evaluating images interactively

The right side of the main window is dedicated to viewing and evaluating the generated images. Locations of images and data files can either be entered directly or copied from the left side.

In the evaluation window, one can scroll through one or two images at once; the scroll bars in both viewing panes are tied to one another. Clicking on any SNP will reveal the data associated with it, which can be either copied to the clipboard or sent to the UCSC Genome Browser to view the immediate vicinity (±10 kb) of the selected SNP.

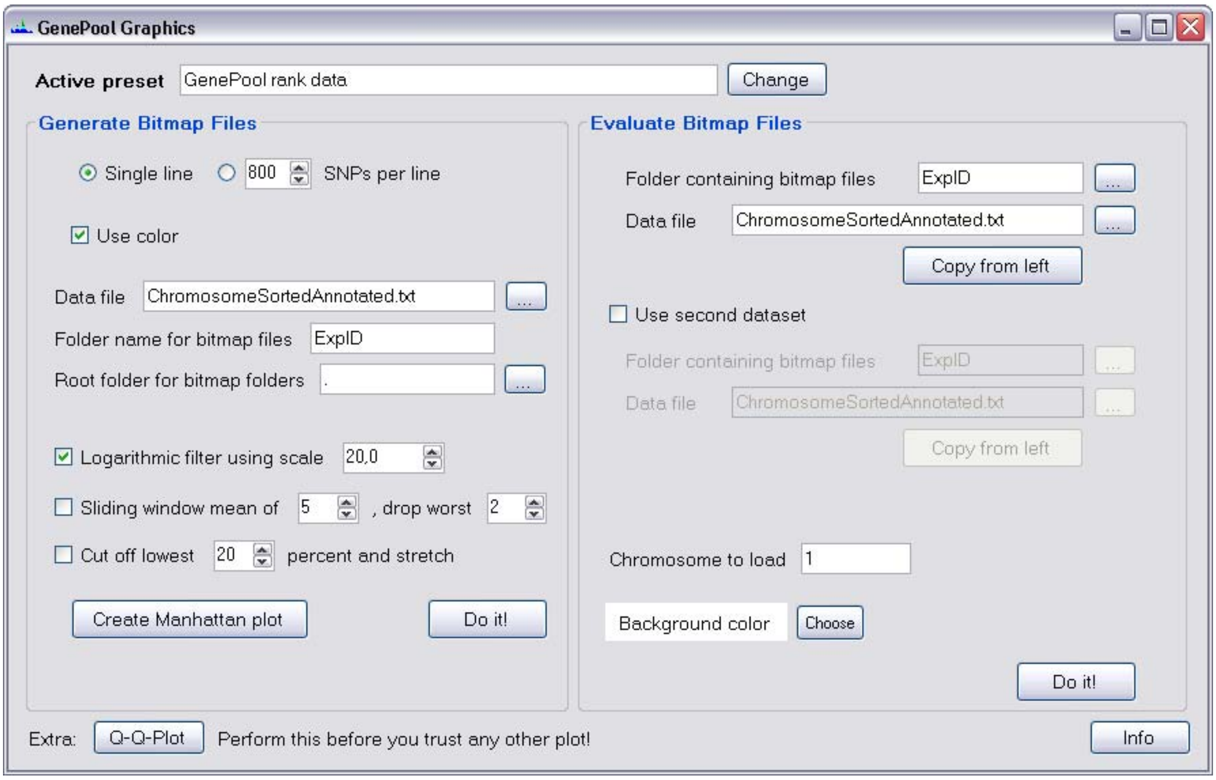

The main window of GPGraphics. Various options for generating images are within the left panel; the viewing options are on the right. In the bottom left corner, the button to launch the QQ-plot module.

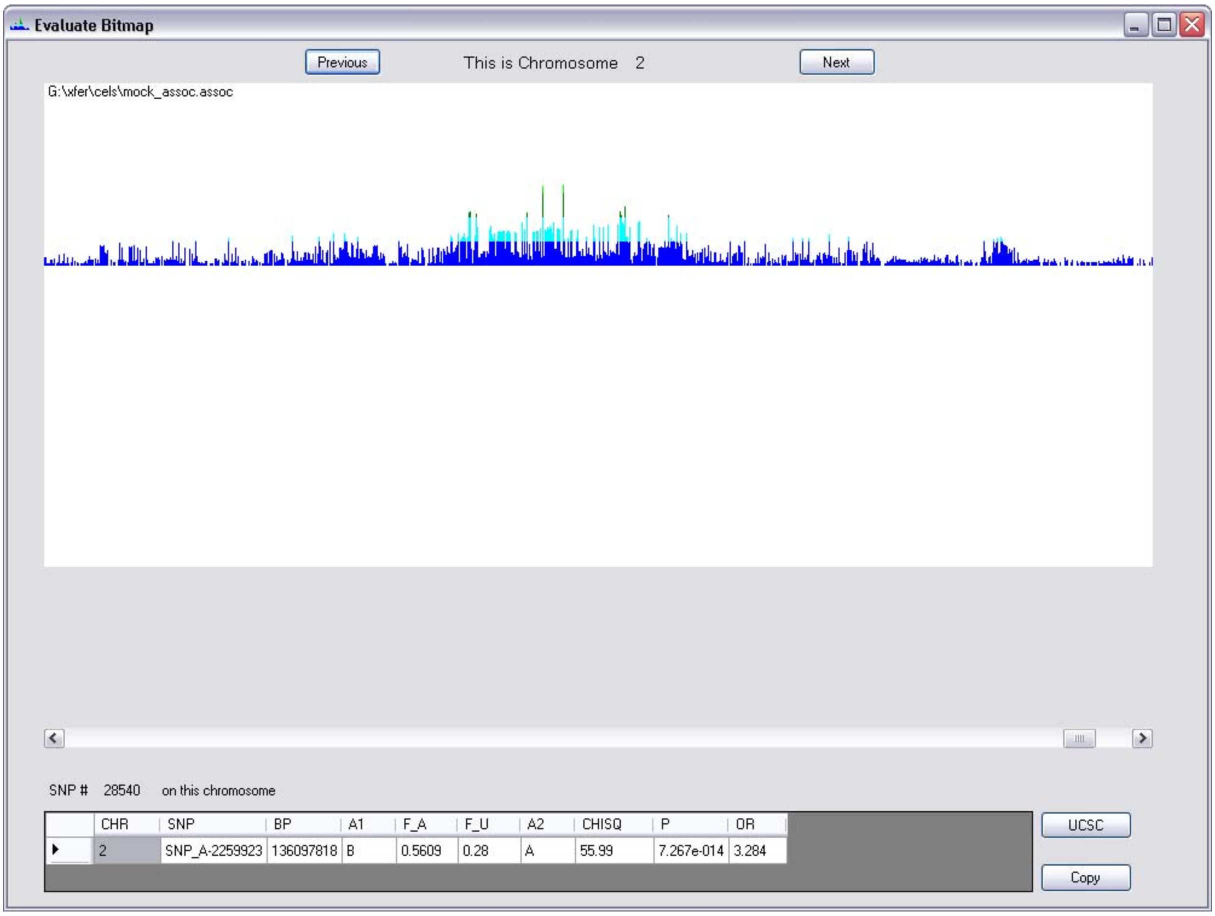

The evaluation window. Either one or two data sets may be viewed at once. SNP results can be copied to the clipboard or looked up using the UCSC genome browser.

The QQ-plot module

GPGraphics also has a module to generate a quantile-quantile plot of expected vs. observed p-values. If the data are from a 2df-chi-square test, the genomic inflation factor and corrected p values can also be calculated besides the theoretical quantiles.

If a file is already sorted on p value (lowest first), there is the option to just add the theoretical quantiles as an additional column.

A file which already has all necessary data (such as PLINK's .assoc.adjusted files) may be directly used as input in step 2.

The user has to select the desired scale of the y axis, which will depend on the lowest expected p-Values.

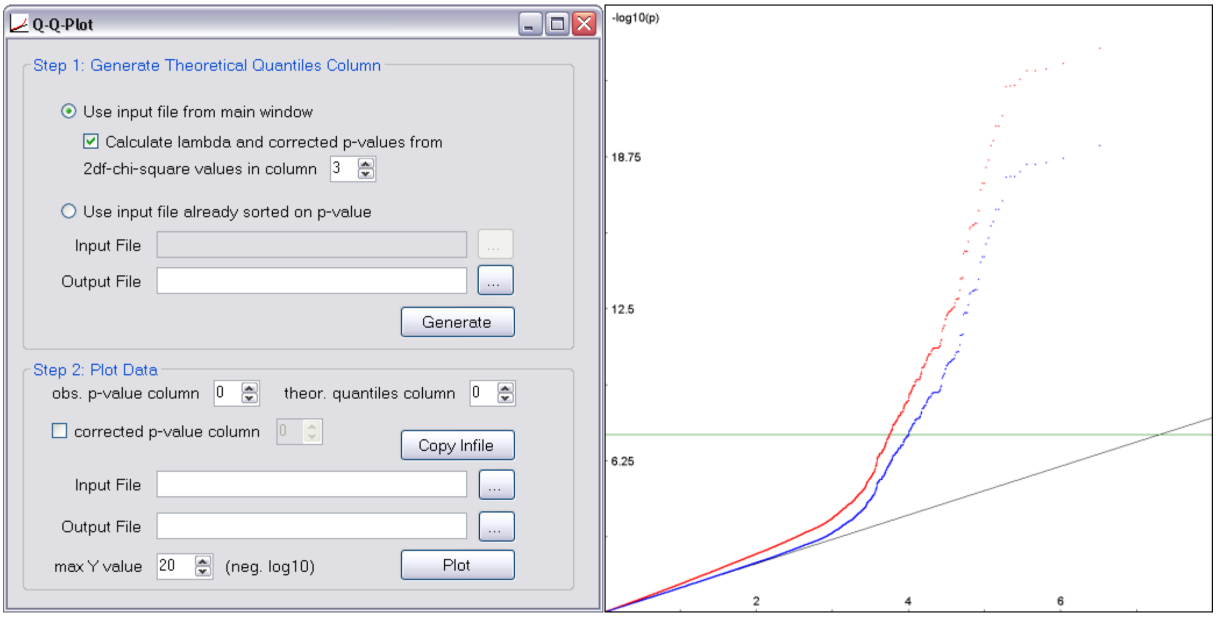

QQ plot module of GPGraphics and its output. Uncorrected p-values are shown in red, GC corrected values in blue. The green line is the genome wide significance threshold of 5E-08.

References

The GenePool Software and manual from TGen are available here:

http://genepool.tgen.org/

GenePool was originaly published as:

Pearson JV, Huentelman MJ, Halperin RF, Tembe WD, Melquist S, Homer N, Brun M, Szelinger S, Coon KD, Zismann VL, Webster JA, Beach T, Sando SB, Aasly JO, Heun R, Jessen F, Kolsch H, Tsolaki M, Daniilidou M, Reiman EM, Papassotiropoulos A, Hutton ML, Stephan DA, Craig DW. Identification of the genetic basis for complex disorders by use of pooling-based genomewide single-nucleotide-polymorphism association studies. Am J Hum Genet. 2007 Jan;80(1):126-39.
