# Supplementary material for: GPFrontend and GPGraphics: graphical analysis tools for genetic association studies
Source: BMC Bioinformatics. 2010 Sep 21;11:472. doi: 10.1186/1471-2105-11-472 (PMC2949897; doi:10.1186/1471-2105-11-472)
Supplement: Additional file 2 — Tutorials for GPFrontend and GPGraphics. Three step-by-step tutorials with actual data for both pooling based studies and single sample GWAS [file 1471-2105-11-472-S2.PDF]

GPFrontend and GPGraphics Tutorials

This document contains several step-by-step tutorials using actual experimental data. It demonstrates the usability of GPGraphics for a variety of different data formats. The tutorials are:

1. Pooling-based association study of pseudoexfoliation syndrome

2. Genome-wide association study (GWAS) in psoriatic arthritis

2a. Quantile-quantile plot generation for the same study

Tutorial 1: Pooling-based association study of pseudoexfoliation syndrome

Background: Three pools of patients with pseudoexfoliation syndrome (PEX) were analyzed using the Affymetrix 500k SNP Array. The pools are called pool 8 (containing 80 PEX patients), pool 9 (containing 80 pseudoexfoliation glaucoma patients) and pool 11 (containing 39 PEX patients who had been genotyped at the LOXL1 locus (known to confer risk of glaucoma[1]) and found to carry a non-risk haplotype.

As the Affymetrix 500k Array is divided into an Nsp and a Sty chip, we get six CEL files from our three pools.

Starting GPFrontend, we first select “Use gpextract”. We now add the case CEL files by clicking the “Add” button on the case side. After navigating to the folder containing the CEL files, we see all six of them:

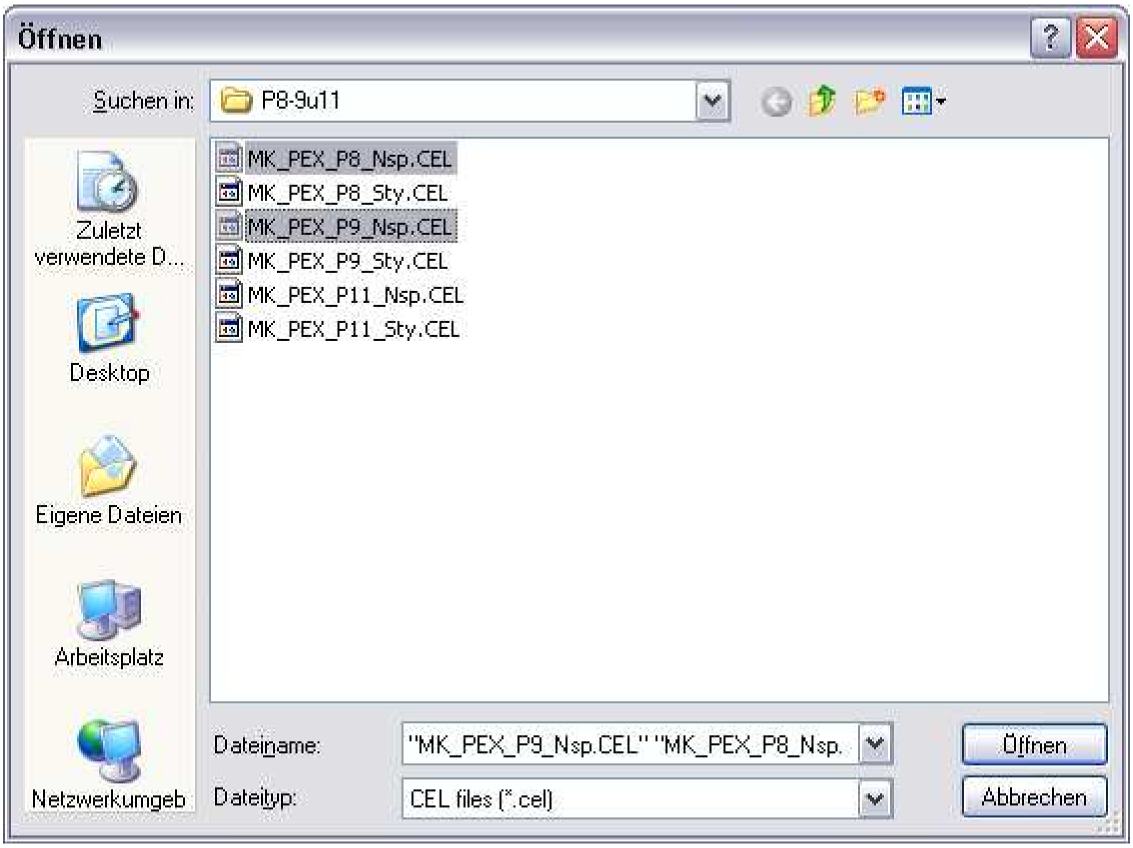

We now select the Nsp files for pool 8 and pool 9 (holding down the Control key while clicking). After clicking “Open”, the files appear in our CEL file list on the cases side. We do the same with MK_PEX_P11_Nsp.CEL on the controls side. We now need to select a CDF file (provided by Affymetrix) that defines the array contents. Accordingly, Mapping250K_Nsp.CDF is chosen and Nsp selected as the relevant enzyme. Finally, we rename the experiment file (generated during extraction) to something more meaningful. The screen now looks thus:

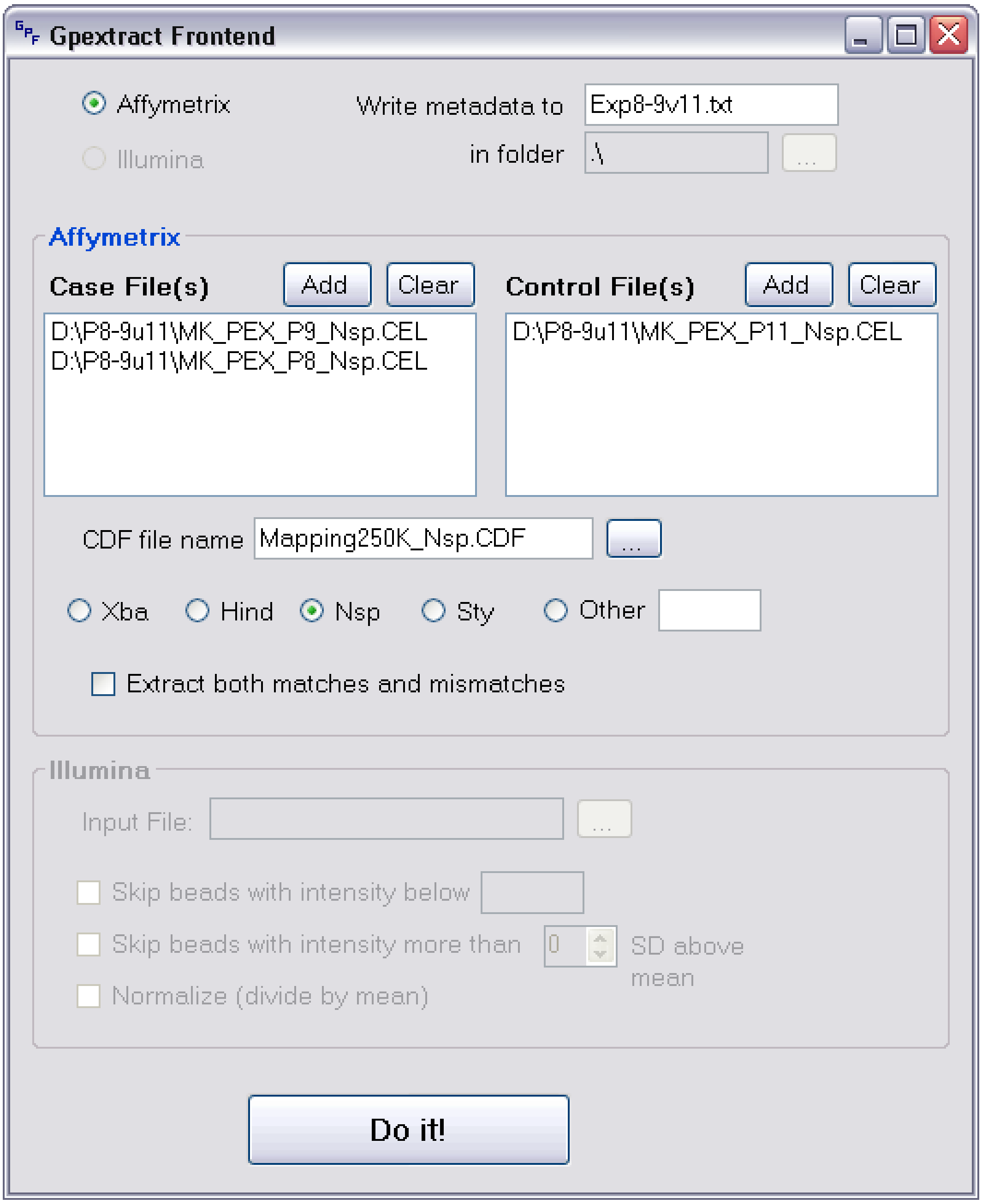

Clicking on "Do it!" will perform the extraction for the Nsp files. After clearing the CEL file lists ("Clear" buttons), we now do exactly the same steps for the Sty files, also choosing the appropriate CDF file. The experiment file must not be renamed at this point, as the metadata for the Sty files are appended to it.

The dialogue should now look just as in the following figure. Another click on "Do it" will perform the second extraction, and the extracted data files are ready for further analysis.

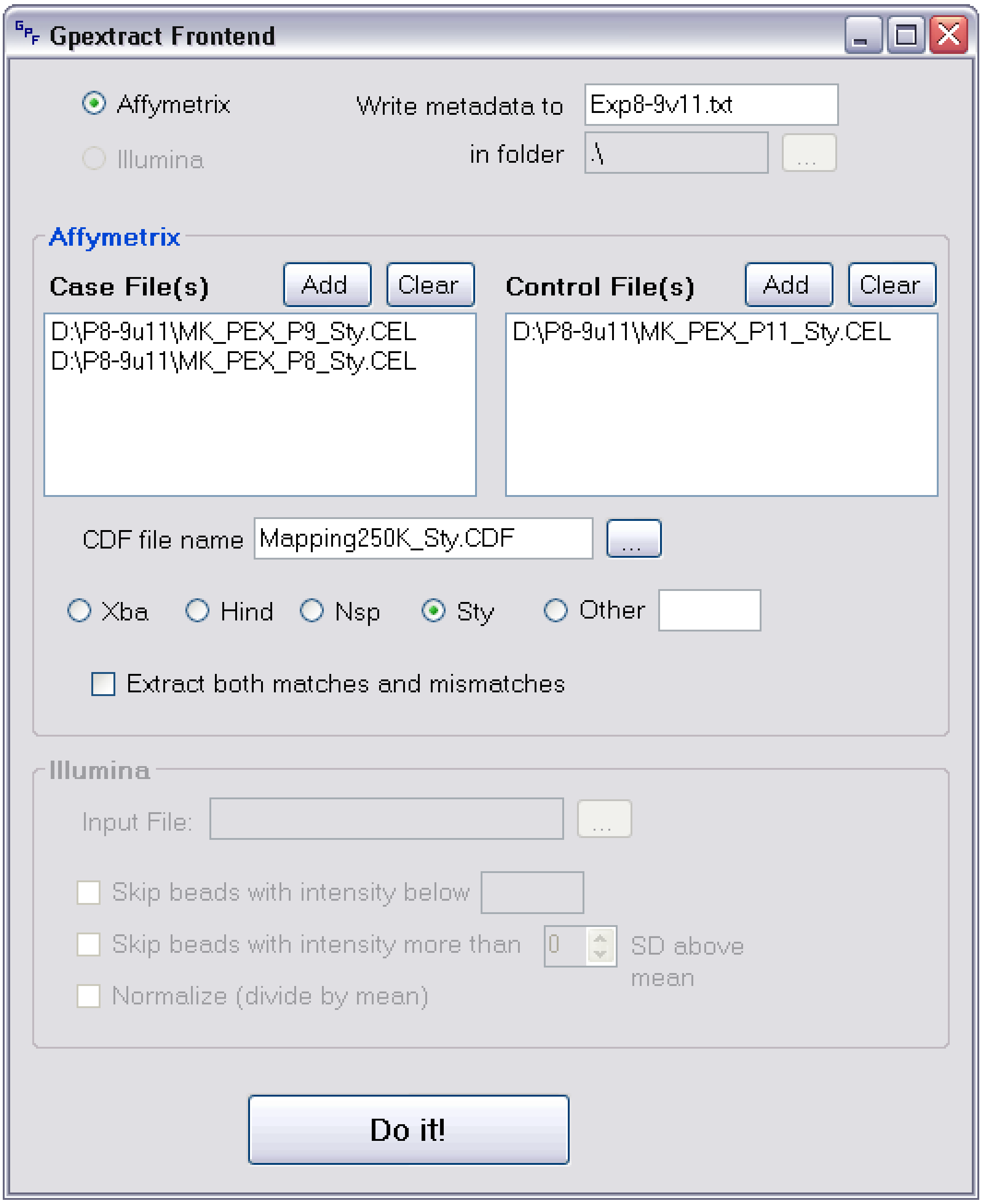

We are now ready to use the gpanalyze frontend to perform an actual statistical analysis of the extracted data. We close the Gpextract Frontend dialogue and bring up the frontend for gpanalyze by clicking "Use gpanalyze" in the main window of GPFrontend.

We now have a wide variety of statistical options to choose from. Since a silhouette score, the algorithm selected by default, needs more than one sample file in both groups (cases and controls), and we only have pool 11 as a control, we need to choose a regular T-test as our algorithm for analysis. We then need to select the experiment file generated in the previous step, an annotation file containing names and positions of SNPs on the array (easily generated from Affymetrix annotation files, but also available from the author of this paper upon request), and an output

folder for the files generated by the analysis. Furthermore, an experiment prefix of up to five characters can (and in fact, should) be stated, so the file names become unique.

After entering all this, the dialogue now looks as follows:

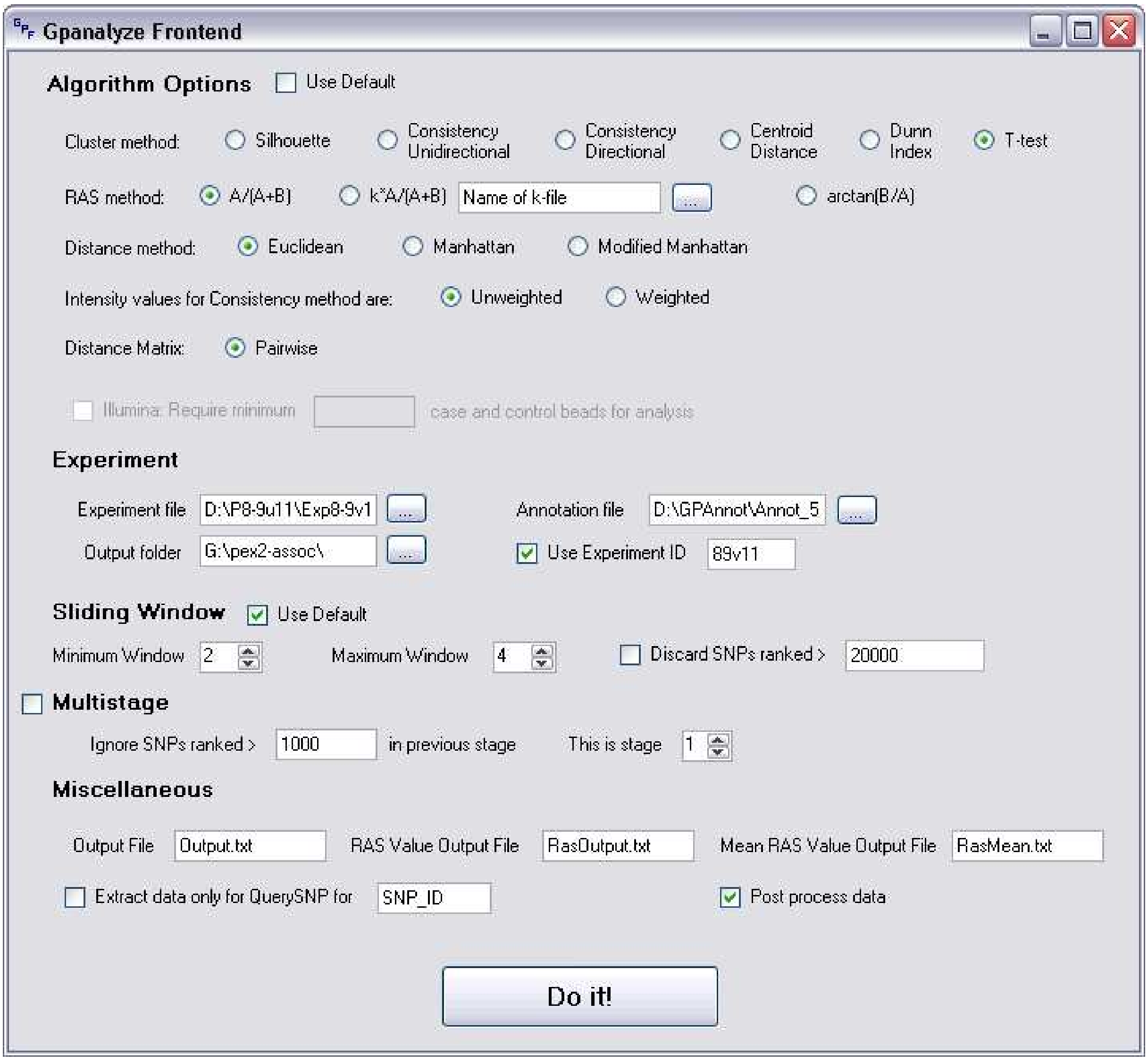

Clicking on “Do it!” will perform the analysis, writing a set of files into the selected output folder. The most important one of these files, and the one analyzed with GPGraphics, is 89v11ChromosomeSortedAnnotated.txt (containing the prefix we have entered before).

We now close GPFrontend and continue our analysis in GPGraphics. In the main dialogue, we first choose “GenePool rank data” from the list of presets, as this is what we want to see. After choosing the aforementioned file 89v11ChromosomeSortedAnnotated.txt, we enter a name for the folder that will contain the generated images, as well as a folder in which the image folder(s) will be. For the moment, no filters are selected:

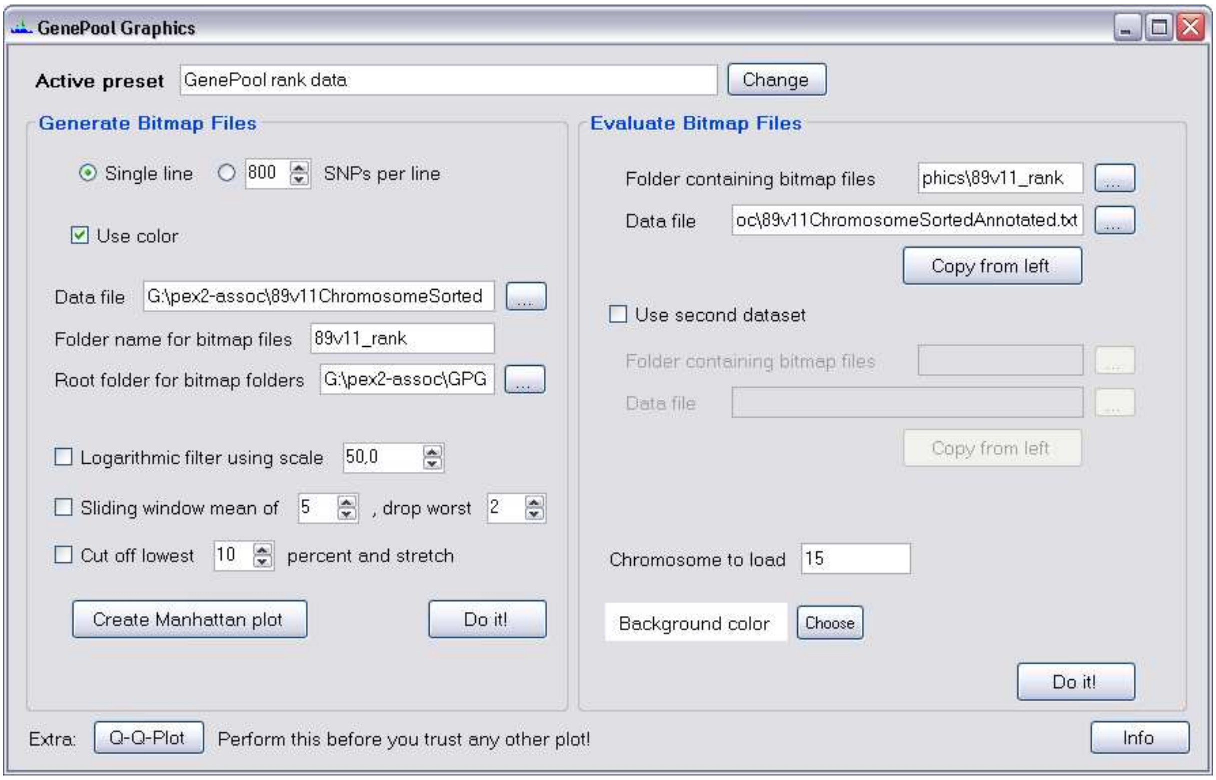

Clicking “Do it!” will generate plots according to the rank of the SNPs’ T-test values. Clicking “Copy from left” on the evaluation side will copy all the necessary information, so we can click “Do it!” to see the data:

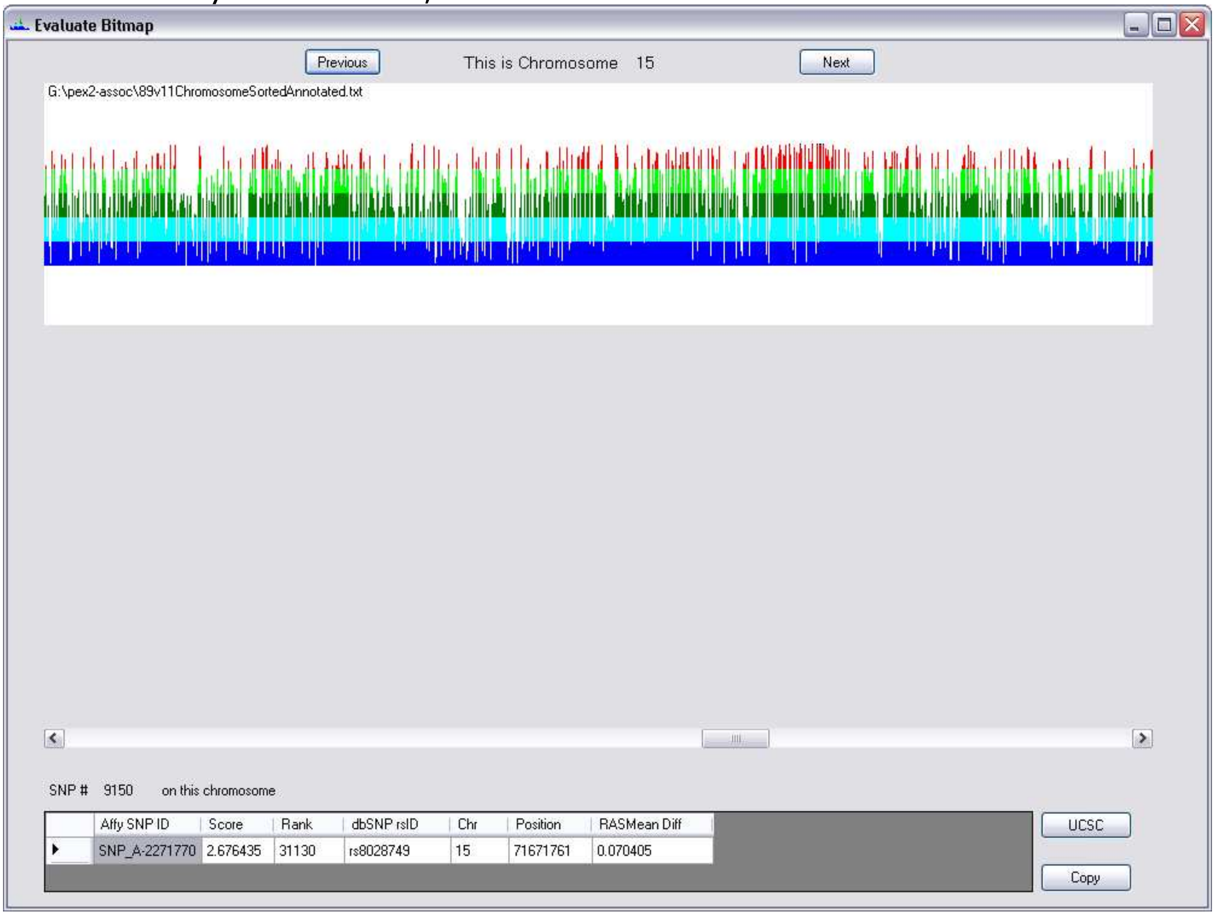

Clearly, this is much too noisy to discern any actual signal of association. So, we close the evaluation window and choose some filter options, taking care to also change the name of the image folder:

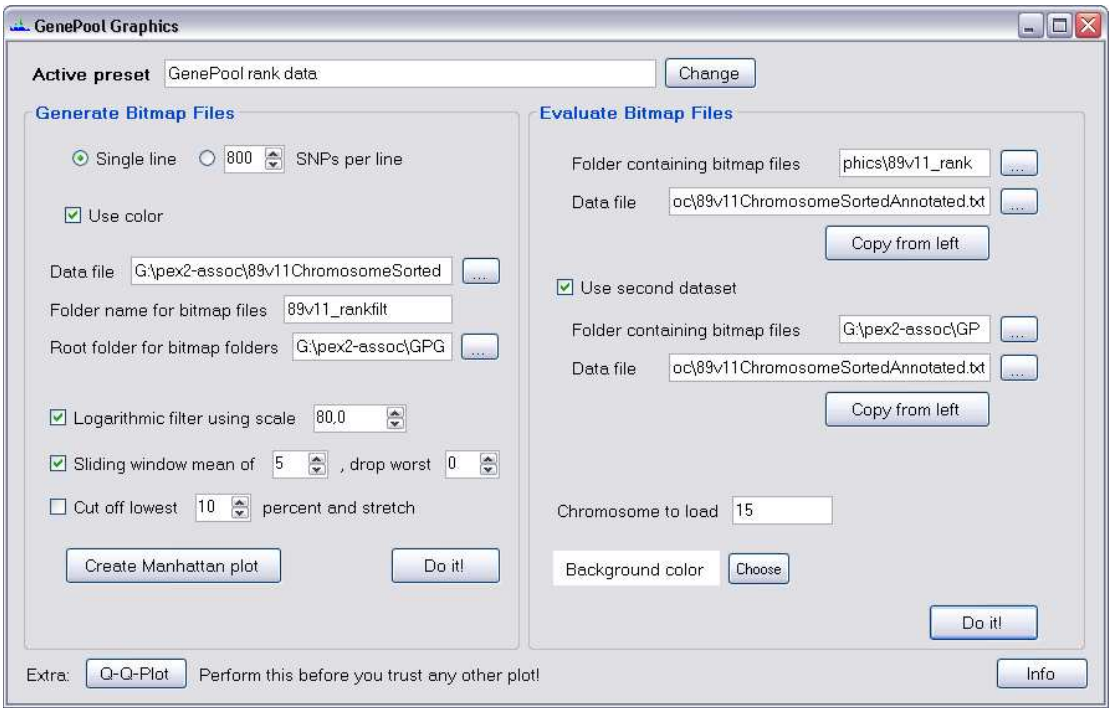

After creating the images (left “Do it!”), we copy the new file information into the second dataset panel and choose to view chromosome 15. What we see now looks much better:

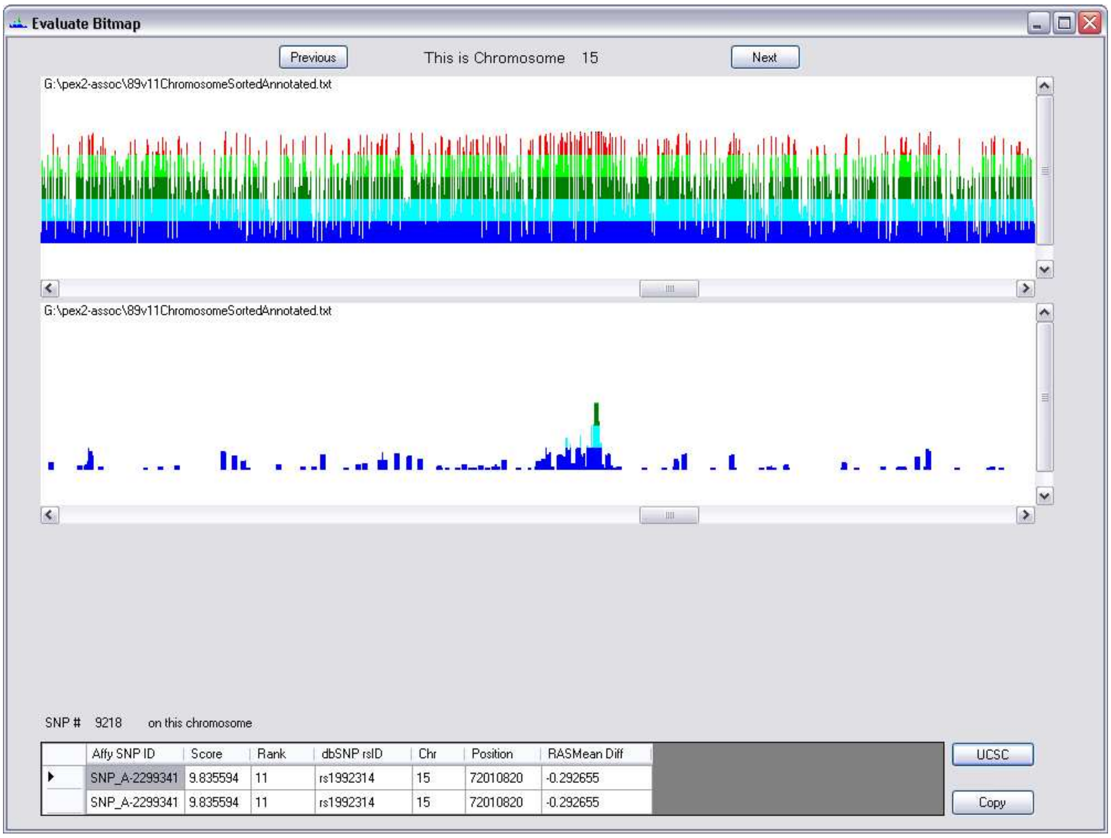

In the filtered view, there is a clearly discernible peak. Clicking on the SNP with the highest signal, we see that there is indeed a perceivable difference in mean RAS values at this position. Clicking the "UCSC" button brings up a browser window showing the locus we selected:

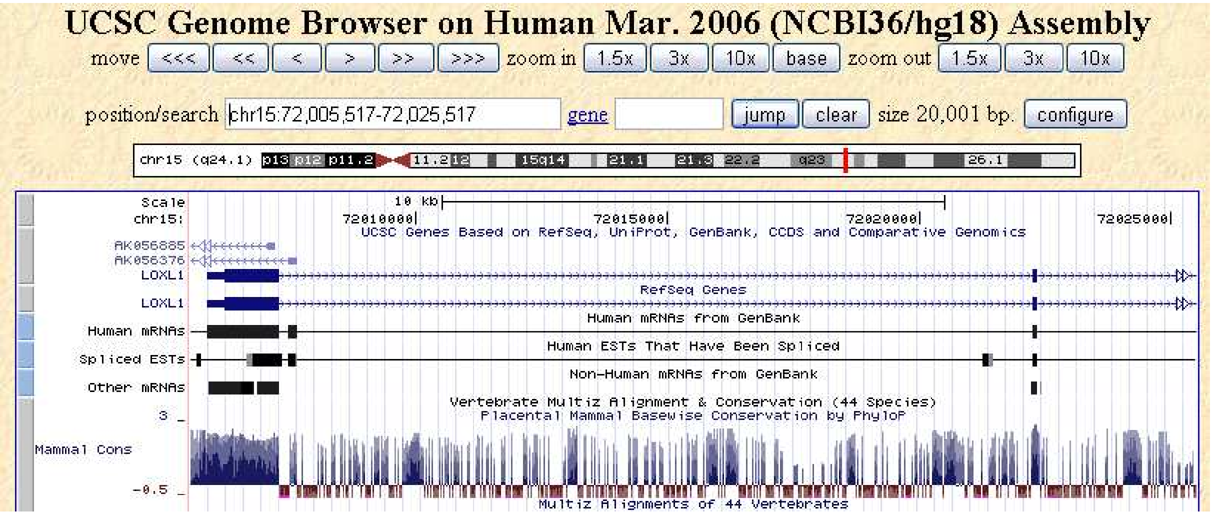

The signal was indeed the LOXL1 locus, which was to be expected given the data set we entered. This, however, provides a good positive control for our entire pooling experiment.

Tutorial 2. Genome-wide association study (GWAS) in psoriatic arthritis

Background: 572 psoriatic arthritis patients and 888 population based controls were genotyped on the Affymetrix 6.0 SNP array; genotypes were called using Affymetrix Power Tools. The data set was imputed to the HapMap data set using MACH1[2] and the most likely imputed genotypes were analyzed with EIGENSTRAT[3].

We now view the unified EIGENSTRAT data, which has been copied into a file called psa-impfilt.txt, using GPGraphics. Having selected "EIGENSTRAT chi-square p-value" from the presets list, we can proceed to load the file and choose a folder for the images as before:

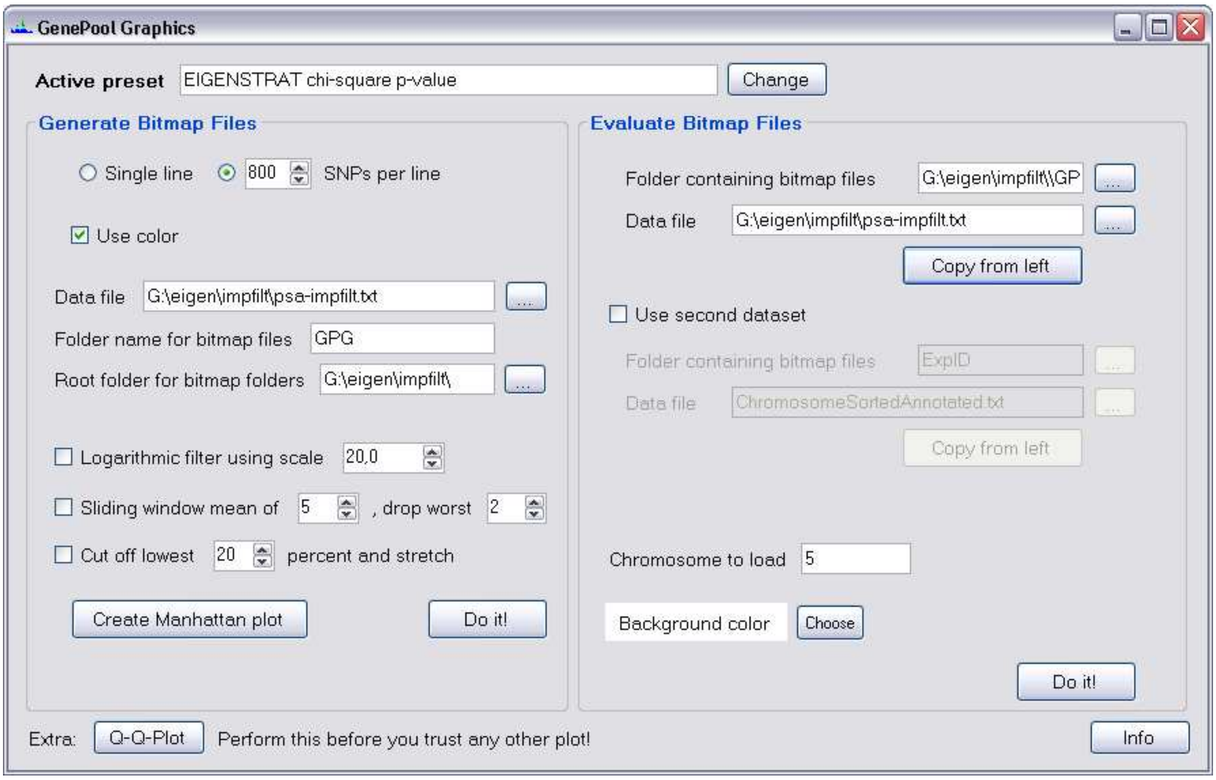

Clicking the left "Do it!" button will generate the images. As this is a GWAS using one array per individual, our data should not be very noisy. Hence, selecting a filter is not recommended in this situation. Copying the file locations to the right ("Copy from left" button), we can now proceed to view chromosome 5:

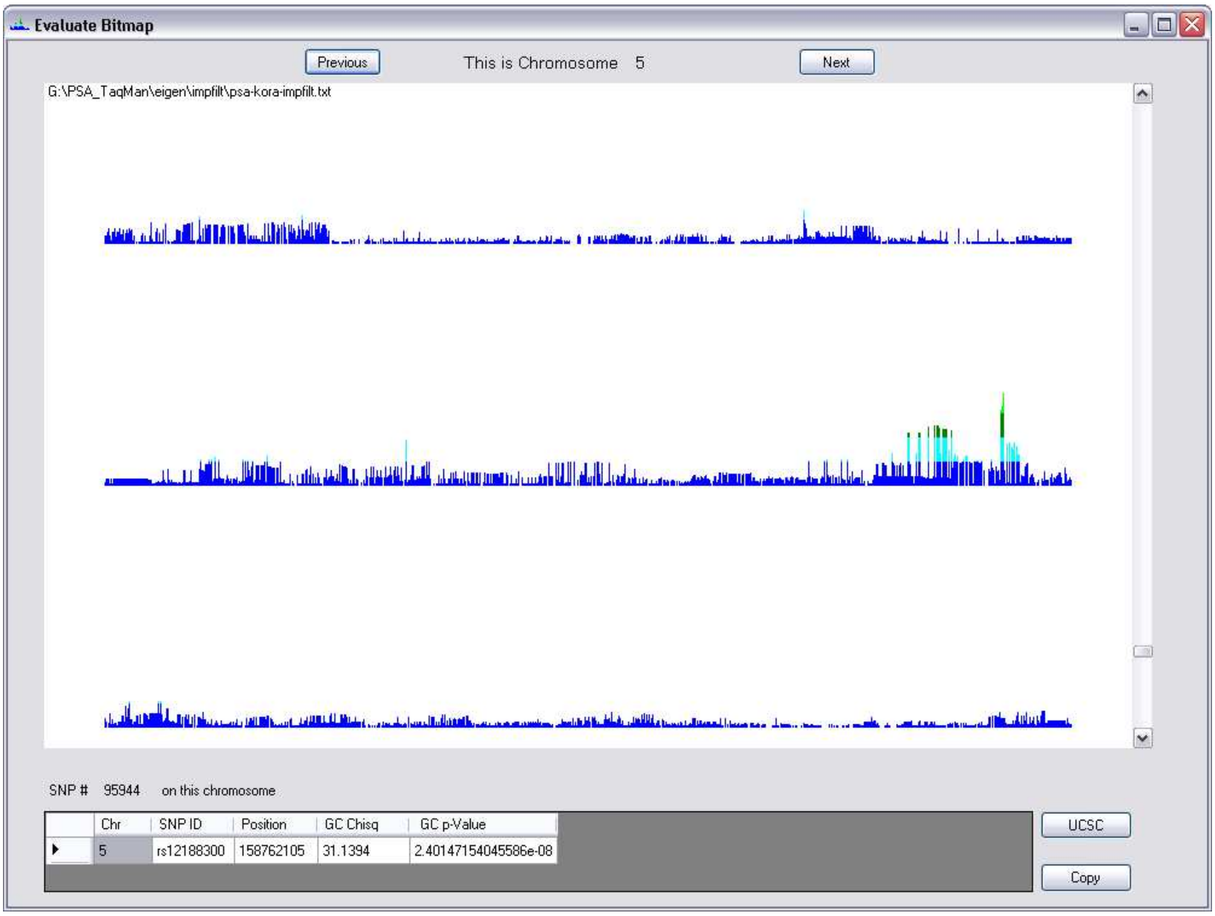

We see a clearly visible peak which, upon closer examination, even turns out to be of genome wide significance (p < 5E−8). Clicking the "UCSC" button reveals that this locus is the IL12B gene, which has previously been reported to be associated with psoriasis[4].

To get a genome wide overview of all genotyped SNPs, a so-called Manhattan plot is often a desired choice. By clicking the "Create Manhattan Plot" button in the main dialogue, we can do just that. After being prompted for a file name and location, the program will create a PNG file with the plot:

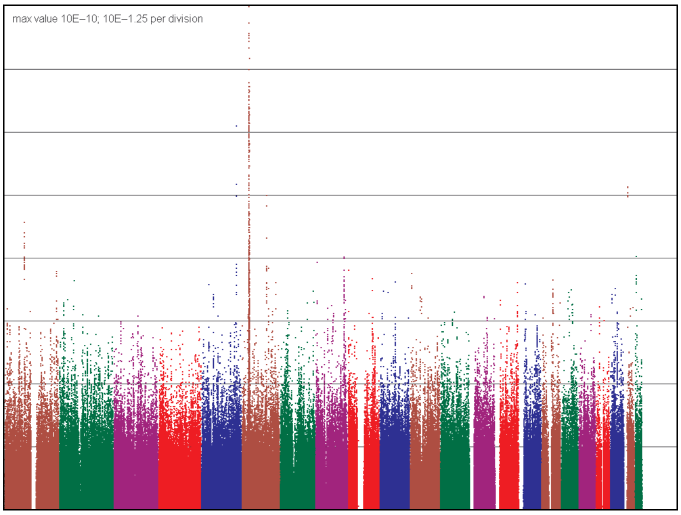

We see many potentially promising loci, most prominently the MHC region on chromosome 6, which has been shown to be associated with psoriasis and psoriatic arthritis on multiple occasions[5].

Tutorial 2a: Quantile-quantile plot generation for the psoriatic arthritis study

In order to see just how far the best p-values deviate from the ones expected under the null hypothesis, a so-called quantile-quantile plot is often used. This type of plot shows the p-values observed in the study – from highest to lowest – plotted against the range of p-values expected by pure chance, i.e., if there is no association. If the best observed p-values differ much from the expected ones, they are much more likely to be truly significant.

We can actually keep the input data we have used for the GWAS and the Manhattan plot. Clicking the "QQ-Plot" button on the bottom of the main window will bring up the QQ-plot module. Here, we select "Use input file from main window". As the EIGENSTRAT values in question are already GC-corrected[6], we need not generate these values. After selecting an output file name and clicking "Generate", a file with sorted p-values and theoretical quantiles is generated:

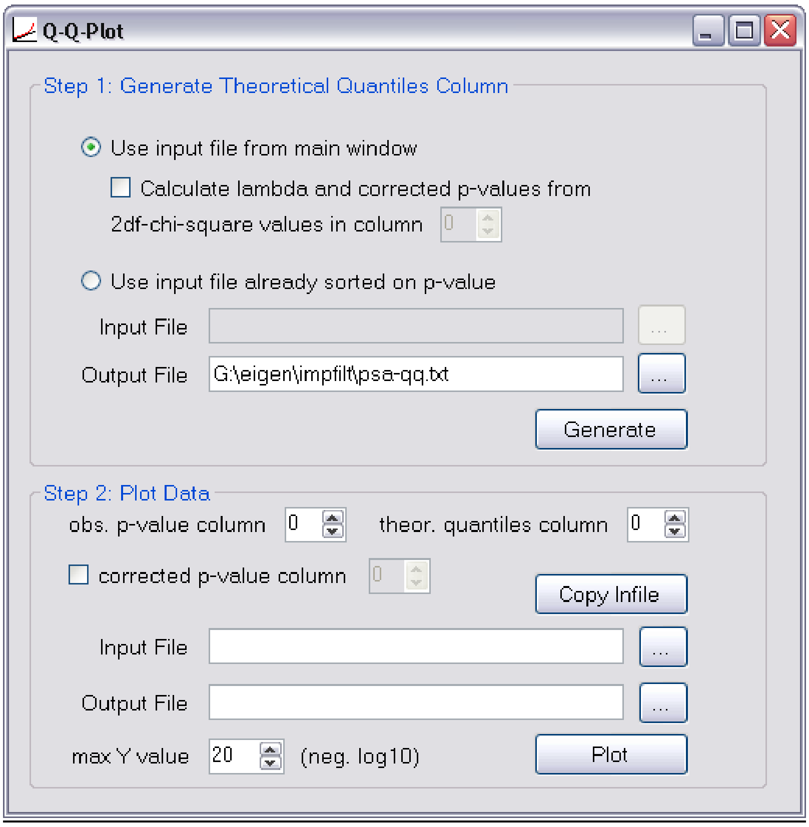

We can now simply copy the path to this file by pressing “Copy Infile”. A closer look at the generated file reveals the observed p-values to be in column 1 (counting from 0) and the expected quantiles in column 2. Since we know the MHC region to be highly associated, we choose the maximum expected Y-(i.e., observed) value to be 10E–20. After selecting an output file name, we are ready to generate the plot:

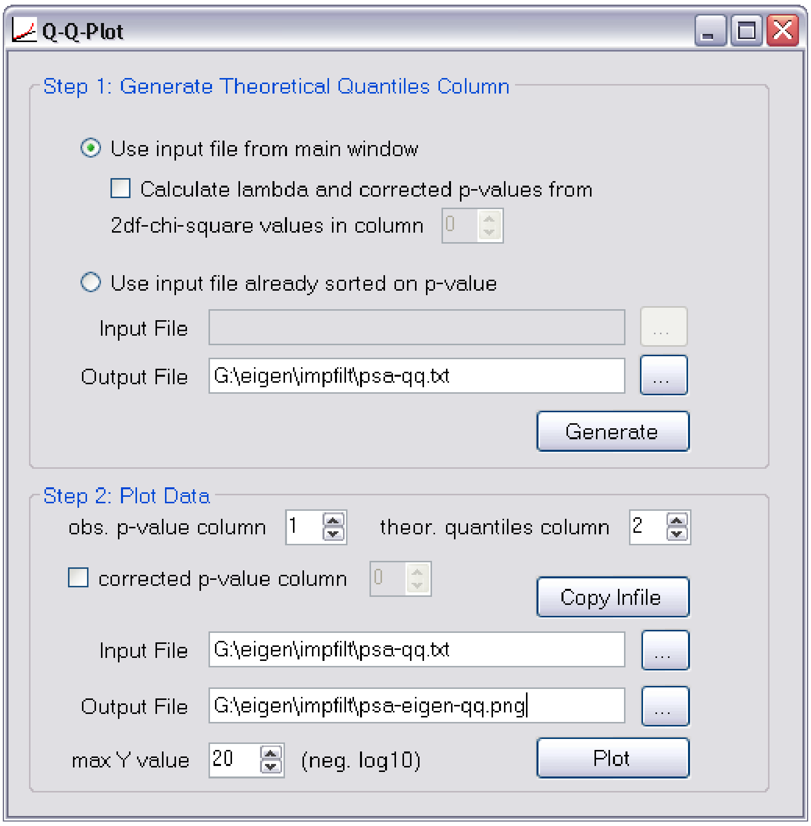

Again, the actual plot is a PNG file, showing the expected quantiles as a solid black line, the observed p-values as a line composed of blue dots, and the genome-wide significance level as a thin green line.

We see a strong deviation from the expected quantiles, which is no surprise given the huge number of strongly associated SNPs in the MHC region. The fact that the lower part of the blue line correlates closely with the expected quantiles shows that the GC correction was apparently successful.

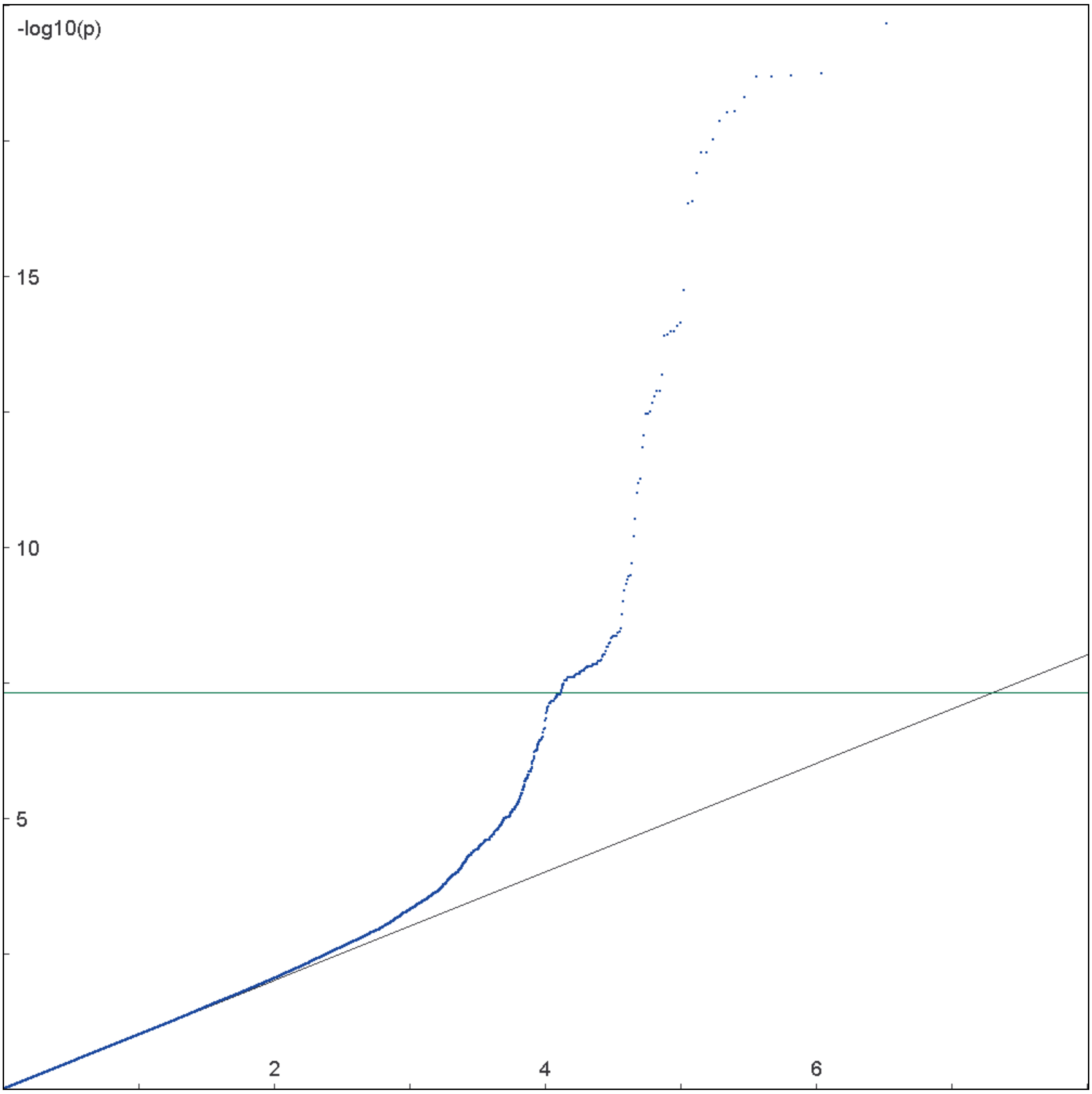
-log10(p)
15
10
5
2
4
6

1. Thorleifsson G, Magnusson KP, Sulem P, Walters GB, Gudbjartsson DF, Stefansson H, Jonsson T, Jonasdottir A, Stefansdottir G, Masson G, et al: **Common sequence variants in the LOXL1 gene confer susceptibility to exfoliation glaucoma.** *Science* 2007, **317:**1397-1400.
2. Li Y, Willer C, Sanna S, Abecasis G: **Genotype imputation.** *Annu Rev Genomics Hum Genet* 2009, **10:**387-406.
3. Price AL, Patterson NJ, Plenge RM, Weinblatt ME, Shadick NA, Reich D: **Principal components analysis corrects for stratification in genome-wide association studies.** *Nat Genet* 2006, **38:**904-909.
4. Tsunemi Y, Saeki H, Nakamura K, Sekiya T, Hirai K, Fujita H, Asano N, Kishimoto M, Tanida Y, Kakinuma T, et al: **Interleukin-12 p40 gene (IL12B) 3'-untranslated region polymorphism is associated with susceptibility to atopic dermatitis and psoriasis vulgaris.** *J Dermatol Sci* 2002, **30:**161-166.
5. Bowcock AM, Krueger JG: **Getting under the skin: the immunogenetics of psoriasis.** *Nat Rev Immunol* 2005, **5:**699-711.
6. Devlin B, Bacanu SA, Roeder K: **Genomic Control to the extreme.** *Nat Genet* 2004, **36:**1129-1130; author reply 1131.
